# RIP3 is an upregulator of aerobic metabolism and the enhanced respiration by necrosomal RIP3 feeds back on necrosome to promote necroptosis

**DOI:** 10.1038/s41418-018-0075-x

**Published:** 2018-03-09

**Authors:** Xingfeng Qiu, Yingying Zhang, Jiahuai Han

**Affiliations:** 1 Department of Gastrointestinal Surgery, Zhongshan Hospital of Xiamen University, Xiamen, Fujian China; 2 State Key Laboratory of Cellular Stress Biology, Innovation Center for Cell Signaling Network, School of Life Sciences, Xiamen University, Xiamen, Fujian China

Necroptosis is a type of programmed cell death with necrotic morphology, occurring in a variety of biological processes, including inflammation and host defense upon viral infection. The current nomenclature defines necroptosis as cell death mediated by signaling transduction from receptor interacting protein (RIP) 1 to RIP3 [1]. However, RIP3-dependent cell death would be a more precise definition of necroptosis, because RIP3 is indispensable for necroptosis but RIP1 is not involved in the signal transduction in some cases [2], and deletion of RIP1 even promotes RIP3-mediated necroptosis under certain conditions [3]. Tumor necrosis factor-α (TNF)-induced necroptosis requires the signaling transduction from RIP1 to RIP3. RIP1–RIP3 interaction permits RIP3 to recruit more RIP3, which leads to RIP3–RIP3 homo-oligomerization/aggregation and RIP3 autophosphorylation. Phosphorylated RIP3 in the aggregate (complex) recruits and phosphorylates mixed lineage kinase domain-like (MLKL). This RIP3-containing complex is called necrosome or ripoptosome, on which the phosphorylated MLKL was oligomerized, followed by MLKL oligomer translocation to the plasma membrane to execute necroptosis [4].

Reactive oxygen species (ROS) participate in the regulation of necroptosis in many but not all cell types and the function of ROS in necroptosis is to enhance necrosome formation [5]. A recent study from our laboratory demonstrated that ROS activate RIP1 autophosphorylation via oxidation of three specific cysteines in RIP1, promoting the formation of RIP3 homo-aggregates [6], a key step in functional necrosome assembly. Our early study showed that TNF-induced ROS production is RIP3 dependent, and the metabolic enzymes glycogen phosphorylase (PYGL), glutamate-ammonia ligase (GLUL), and glutamate dehydrogenase 1 (GLUD1) are activated by RIP3, resulting in enhancement of aerobic respiration and thus likely contributing to TNF-induced ROS [7]. However, the other and perhaps major mechanism(s) still await(s) being discovered.

In the current issue of *Nature Cell Biology*, Yang et al. [8] from our laboratory report that pyruvate dehydrogenase complex PDC (also known as PDH) pyruvate dehydrogenase (PDH), the rate-limiting enzyme linking glycolysis to aerobic respiration, can be activated by RIP3 and the enhancement of aerobic respiration leads to more ROS production [8]. The study was initiated by analyzing the correlation among the levels of aerobic respiration (oxygen consumption rate (OCR)), ROS production, and necroptosis in TNF-induced necroptosis, and found that the induction of OCR and ROS is RIP3 dependent and well correlates with necroptosis. The authors then used mitochondrial respiration inhibitors and mitochondrial depletion to show that TNF-induced increase of aerobic respiration is responsible for ROS induction in necroptosis.

By using metabolic labeling and medium depletion of glutamine, the authors demonstrated that glutamine catabolism by GLUL and GLUD1 contributes to TNF-induced increase of aerobic respiration but is not the major contributor. The authors then proceeded to investigate other targets of RIP3 in regulating aerobic respiration. The authors studied PDC, because PDC-E1β subunit was found in RIP3 immunocomplex from TNF-treated cells and PDC is the key enzyme complex that converts pyruvate to acetyl-CoA linking glycolysis to respiration. The authors utilized inhibitors and gene knockdown or knockout strategies to reveal that blocking PDC but not lactate dehydrogenase, i.e., for anaerobic fermentation, inhibits TNF-induced OCR increase, ROS production, and necroptosis occurrence. In addition, depletion of pyruvate, inhibition of pyruvate transport into the mitochondria, or inhibition of pyruvate carrier proteins in the mitochondria can all inhibit TNF-induced necroptosis. Collectively, these data demonstrate that PDC-mediated catalysis of pyruvate leads to aerobic respiration elevation and the subsequent necroptosis.

Next, the authors provided the mechanism of how RIP3 regulates PDC. Metabolic labeling and enzymatic assays showed that RIP3 directly increases PDC activity. The regulation of PDC activity by RIP3 is different from the known dephosphorylation mechanism. The authors found that RIP3 interacts with PDC and activates PDC by phosphorylating PDC-E3 subunit on threonine 135. Furthermore, the authors found that the recruitment of MLKL to necrosome during necroptosis is required for RIP3 to enhance aerobic respiration. The most likely role of MLKL in this process is that MLKL is required for RIP3-containing necrosome to translocate to the mitochondria where RIP3 can interact with and phosphorylate PDC. Thus, it is the RIP3 in the MLKL-containing necrosome that can activate PDC. Given the fact that PYGL is also a target of RIP3, converting glucose catabolism to aerobic respiration can be enhanced at multiple sites by RIP3.

**Fig. 1:**
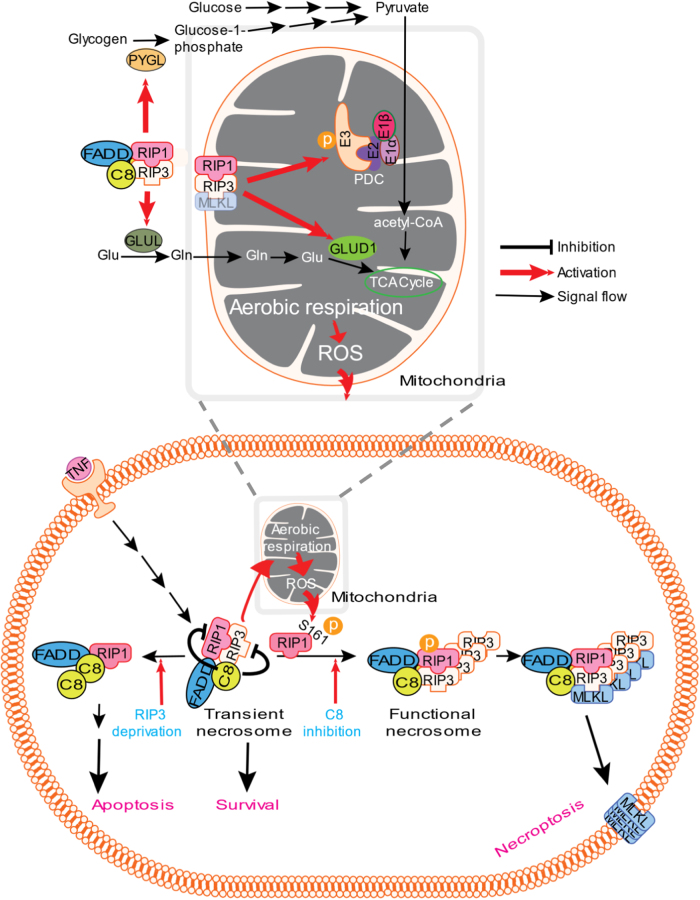
Model of TNF-induced cell death. In most types of cells FADD, caspase-8 (C8), RIP1, and RIP3 form complex (necrosome) upon TNF stimulation. Necrosome at this stage is transient due to that C8 cuts RIP1 and RIP3, and thus no death signal is transduced. Inhibition of C8 stabilizes necrosome and thus allows the signal to flow toward necroptosis. This News & Views focuses on another driving force—RIP3-mediated enhancement of aerobic respiration—which pushes necroptosis-prone cells towards necroptosis. RIP3 in either cytosol- or mitochondrion-associated necrosome is involved in the activation of metabolic enzymes with different subcellular locations (upper, enlarged schematic diagram). PYGL, PDC, GLUL, and GLUD1 are activated by RIP3, and the activation of PDC contributes more than the others to RIP3-mediated enhancement of aerobic respiration. ROS produced by the enhanced respiration promote the formation of functional necrosome via oxidation of RIP1 and subsequent RIP1 autophosphorylation on serine 161

The aggregate of autophosphorylated RIP3 in necrosome functions as the platform for MLKL recruitment, phosphorylation, oligomerization, and release from necrosome/translocation to plasma membrane. As the amount and/or size of RIP3 aggregates in necrosome determine whether the cell will undergo necroptosis, signals/effectors that promote RIP3 aggregation should drive cells toward a necroptotic cell fate. Besides RIP3, a number of proteins such as FADD and caspase-8 are associated with necrosome in a stimulus-dependent manner. For example, FADD, caspase-8, and RIP1 form complex with RIP3 in TNF-treated L929 cells (Fig. 1), whereas DAI forms complex with RIP3 in RIP1 knockout keratinocytes [9]. In the case of TNF-induced necroptosis, caspase-8 can cut RIP1 and RIP3, resulting in disruption of the complex. We can call this complex as transient necrosome and this necrosome actually functions as survival signal because caspase-8 and RIP3 prevent each other from mediating apoptosis and necroptosis, respectively (Fig. 1). Indeed, many types of cells do not undergo cell death upon TNF stimulation. Necrosomal RIP3-mediated increase of aerobic respiration positively feeds back on necrosome formation via ROS (Fig. 1). RIP3 in necrosome activates cytosolic PYGL and GLUL, which promote glucose and glutamine catabolism, respectively. RIP3 in mitochondrial necrosome activates PDC and GLUD1, which both promote TCA cycle using pyruvate and glutamic acid as energy substrate, respectively (Fig. 1., upper, enlarged schematic diagram). Similar to the inhibition of caspase-8 that has been widely used to promote necroptosis [10], ROS that resulted from the increased aerobic respiration are also a driving force for the formation of functional necrosome in many necroptosis processes (Fig. 1).

Because of the essential role of aerobic respiration in energy metabolism, upregulation of aerobic respiration by RIP3 must have functions other than enhancing necroptosis. However, studies on RIP3 as a metabolic regulator are very limited. RIP3 appears to have a role in hepatic glycogen use in mouse sepsis model [11]; however, besides this work the role of RIP3 in metabolism-related biological processes has not been reported. Thus, RIP3 should not be neglected when a biological process with an increase of aerobic respiration is studied.
